# A Novel ex vivo Snare Tip Soft Coagulation Model for Deliberate Self-Practice and Assessing Endoscopic Tip-Control Competency: The HAM-2 Study

**DOI:** 10.1055/a-2953-6394

**Published:** 2026-09-23

**Authors:** Tamas Tornai, Maria Eva Argenziano, Michele Montori, Pieter Jan Poortmans, Andrea Sorge, Sander Smeets, Lynn Debels, Lobke Desomer, David J. Tate

**Affiliations:** 1 Institute of Pancreatic Diseases 37637 Semmelweis UniversityBudapestHungary; 2 Department of Gastroenterology and Hepatology 60200 University Hospital GhentGhentFlandersBelgium; 3 Medicine and Health Sciences 54498 Ghent University Faculty of Medicine and Health SciencesGhentFlandersBelgium; 4 Clinic of Gastroenterology, Hepathology and Emergency Digestive Endoscopy 9294 Universita Politecnica delle MarcheAnconaItaly; 5 Clinic of Gastroenterology, Hepatology and Emergency Digestive Endoscopy 9294 Università Politecnica delle MarcheAnconaItaly; 6 Department of Gastroenterology and Hepatology 60201 UZ BrusselBrusselsBelgium; 7 Department of Surgery, Oncology and Gastroenterology 18624 Padua University HospitalPaduaVenetoItaly; 8 Faculty of Medicine and Health Sciences 26656 Ghent UniversityGhentFlandersBelgium; 9 Department of Gastroenterology and Hepatology 192827 AZ Delta vzwRoeselareBelgium

**Keywords:** endoscopy upper GI tract, endoscopic resection (ESD, EMRc, ...), endoscopy lower GI tract, endoscopic resection (polypectomy, ESD, EMRc, ...), quality and logistical aspects, training, RFA and ablative methods, performance and complications

## Abstract

**Background and Aims** Tip control is fundamental to advanced endoscopic procedures but lacks objective assessment tools. We aimed to (1) validate an ex vivo snare tip soft coagulation (STSC) model (HAM-2) by assessing its ability to stratify endoscopists by experience (2) evaluate its efficacy in improving tip control following structured training; and (3) identify baseline predictors of training-related improvement.

**Methods** In this prospective pilot validation study (single-centre, single-arm, pre-/post-design), 13 endoscopists (one expert, seven fellows, five trainees) performed STSC along four standardised 3D-printed shapes on a cooked-ham model. Participants underwent pre-training assessment, two unsupervised, structured self-practice sessions (eight shapes), and post-training assessment. Seven blinded raters (three independent raters per video) evaluated video recordings for their subjective impression of tip control and determined objective metrics (number of correct and incorrect hits) using an online tool. Predictors of improvement were analysed using generalised linear mixed models.

**Results** Thirteen endoscopists performed 104 STSC procedures. At baseline, the expert outperformed fellows and trainees in subjective tip control (95.3 vs. 59.7 vs. 56.3, *p* < 0.001), speed (40.6 vs. 23.6 vs. 19.7 mm/min, *p* = 0.002), and accuracy (98.1% vs. 92.7% vs. 90.6%, *p* = 0.003). Considering the overall cohort, subjective tip control improved by 12.3 (95% CI 6.9–17.7, *p* < 0.001), speed by 5.5 mm/min (3.1–7.9, *p* < 0.001), and hit frequency by 6.1 hits/min (3.7–8.5, *p* < 0.001) post-training. Accuracy did not improve significantly (+1.3%, *p* = 0.085). Lower endoscopist baseline accuracy and speed independently predicted greater improvement in subjective tip control.

**Conclusions** In this single-centre, single-expert pilot, the HAM-2 model stratified endoscopists by experience (exploratory) and, through self-practice on the model, yielded measurable improvements in tip control following repeated practice. HAM-2 offers a low-cost, reproducible platform for structured self-practice and task-specific performance measurement.

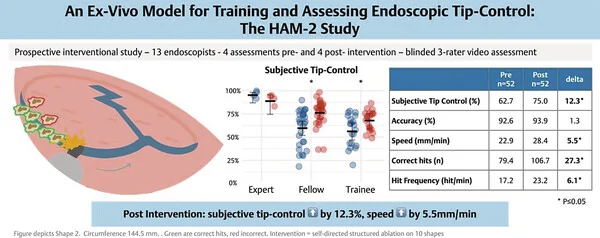

## Introduction

High-quality diagnostic and therapeutic endoscopy requires precise control of the endoscope tip to ensure safe, effective, and efficient procedures. Tip control—the ability to manipulate the distal tip with fine motor precision—is a core skill in endoscopic procedures. Effective tip control is hypothesised to correlate with key procedural outcomes, including accurate lesion targeting, reduced procedural time, and complication avoidance. 1 2 Despite its suggested importance, the assessment and structured training of tip control remain poorly defined.

Current competency-based frameworks in gastrointestinal endoscopy, 3 4 5 6 such as Direct Observation of Procedural Skills and the Global Polypectomy Assessment Tool, provide general evaluations of endoscopic ability. These tools rely on subjective assessments by single evaluators and often lack task-specific, quantitative measures of endoscopic practice such as tip control. Moreover, simulation modalities, including virtual reality simulators and live animal models, are costly, logistically challenging, and generally inaccessible outside high-resource settings. 7 8 9 10 As a result, evaluation of tip control is imprecise and vulnerable to inter-observer variability. 11

Snare tip soft coagulation (STSC) is a precision-dependent endoscopic technique that requires continuous, fine manipulation of the endoscope tip to deliver electrosurgical energy to polypectomy defect margins. 12 STSC performance correlates with endoscopist experience both in vivo 13 and ex vivo, 14 suggesting its potential utility as a surrogate marker for tip-control competency. However, while in vivo assessments provide important insights, they are not scalable for standardisation of training and assessment. Ex vivo alternatives, including the first-generation HAM model, 14 have shown promise but were limited by lack of standardised design, task reproducibility, and objective, blinded scoring criteria.

To address these gaps, we developed a refined ex vivo model (HAM-2) for endoscopic tip control using standardised 3D-printed patterns on a single piece of cooked ham. This model isolates tip control from other procedural variables, offering a reproducible environment for deliberate practice and objective measurement. In this study, we aimed to validate the model and then evaluate the efficacy of unsupervised, structured self-practice using the model in improving both subjective and objective markers of tip-control performance.

## Methods

### Study Design and Setting

This prospective, single-centre pilot study was conducted at the Ghent University Hospital, Belgium, between March and April 2024. The study aimed to validate an ex vivo STSC training model (HAM-2) as a tool for assessing and improving endoscopic tip control. The study was approved by the Institutional Review Board of Ghent University Hospital (ONZ-2024-0214). All participants provided informed consent prior to enrolment.

### Participants

Endoscopists from an interventional tissue resection service at Ghent University Hospital participated. Demographic information, clinical experience (colonoscopies performed, endoscopic mucosal resection [EMR] experience), and years of practice were collected at baseline. Participants were stratified by experience into three groups: experts, interventional fellows, and trainees. Experts were defined as endoscopists with at least 5 years of independent interventional practice and who performed more than 50 EMRs per year. Interventional fellows were those currently enrolled in an advanced endoscopy fellowship programme. Trainees were general endoscopy trainees who had not received any formal advanced endoscopy training.

### Study Aims and Endpoints

The study aimed to validate the HAM-2 model by evaluating its ability to stratify endoscopists according to their level of experience, to assess the efficacy of unsupervised, structured self-practice on the HAM-2 model in improving subjective and objective measures of tip control, and to identify baseline predictors associated with training-related improvement in subjective tip control.

#### Primary Endpoint

- Change in subjective tip-control scores (0–100% scale) between pre- and post-training assessments.

#### Secondary Endpoints

- Changes in objective performance metrics between pre- and post-training assessments:
  - Speed (mm/min)
  - Accuracy (% correct hits)
  - Correct hit frequency (hits/minute)
  - Hit density (hits/cm)
- Stratification of baseline performance by experience level (exploratory)
- Predictors of improvement in subjective tip control after training.
- Inter-rater reliability for all rating measures (correct hits, incorrect hits, subjective tip control).

### Ex vivo Simulation Model (HAM-2)

An endoscopy training model was set up using a standard endoscopy training box (Mobile TD Box, Medical Training Tools, The Netherlands) with a piece of cooked ham placed on the metal plate and kept moist with wet gauze ( Fig. 1A ). A custom-made 3D-printed stencil 15 soaked in blue dye marked four increasingly complex shapes onto the ham, each formed from circles with a radius of 17.25 mm (see Fig. 1B ). Shape details were as follows: **S1** : A single circle, 108.4 mm in length. **S2** : Two intersecting circles, with centres 17.25 mm apart, forming an outer diameter of 51.75 mm and a total length of 144.5 mm. **S3** : Three overlapping circles, adding a third circle on top of Shape 2, with a vertical height of 50 mm and a length of 163.7 mm. **S4** : Two horizontally overlapping Shape 2 arrangements, reaching a height of 50 mm and a length of 176.7 mm. **S0** : A practice shape, 108.4 mm in length, which was a single circle was positioned at the most proximal right side of the ham to allow participants to familiarise themselves with the setup. A Pentax EG-29-i20c gastroscope with an EPK-i8020c processor and a high-definition monitor was used throughout the study.

**Fig. 1:**
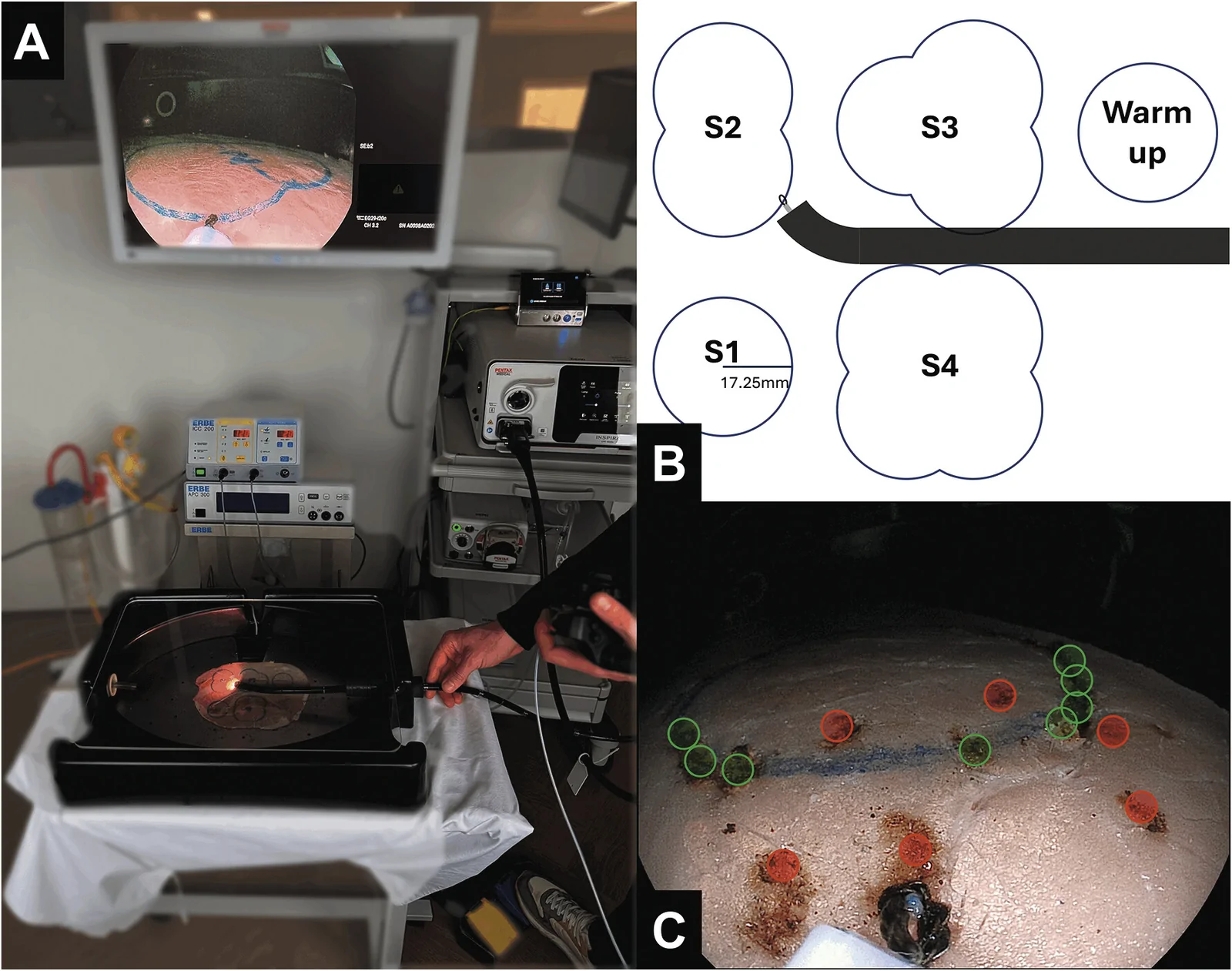
Ex vivo model setup and design. **Panel A** : Standard endoscopy training box (Mobile TD Box, Medical Training Tools, The Netherlands) with a slice of ham placed on the metal plate. An endoscopist is shown performing snare tip soft coagulation using a Pentax EG-29-i20c scope. **Panel B** : Schematic of the four standardised shapes (S1–S4), each formed by circles of 17.25 mm radius, imprinted on the ham; a proportional endoscope is depicted passing into the training box. **Panel C** : Example of correct (green circles) and incorrect (red circles) electrosurgical hits along the imprinted line on the ham, demonstrating how accuracy was assessed in the study.

Model cost: excluding the training box and gastroscope (typically already available in an endoscopy unit), the marginal consumable cost was under €5 per procedure (3D-printed stencil €10–15, once; ham €0.50–1, per procedure; hot snare ≈€15, per five procedures).

### Study Protocol

#### Study Briefing

Before the study, each participant received an explanation of the study rules and expectations, followed by an open discussion to clarify any questions.

#### Pre-training

Participants had 5 min/shape to apply electrosurgical hits sequentially to shapes 1–4, introduced to limit fatigue and simulate real-life endoscopy pressure. If the task was completed before the 5-minute limit, completion time was recorded. Upon completion or at the end of the 5-minute limit, a photograph of the shape was taken directly from above. Each assessment of a single shape was video recorded. The procedure began with the initial application of electrosurgical energy and the timer was paused when cleaning of the endoscope lens or snare tip was necessary. Following reintroduction of the equipment to the simulation box and re-orientation, the timer was restarted when the endoscopist stated they were ready to continue.

#### Unsupervised, Structured Self-practice

After pre-training assessment, each participant had two separate unsupervised self-practice sessions with a mandated structured approach (same as the pre-training assessment) on the ex vivo ham model. These sessions had the same 5-minute time limit for each shape, were scheduled on separate days and were videotaped but not rated.

#### Post-training

After training sessions, participants were reassessed using the same protocol as in the pre-training phase. These procedures were also video recorded. To ensure unbiased rating, videos did not contain any features, images or information that would reveal the identity of the performing endoscopist. Subsequently blinded raters evaluated the recordings.

### Rating



**Video 1** HAM2 set-up, high-level performance on shape 1 and low-level performance on shape 3.


Seven endoscopists from the interventional service served as blinded raters (two experts, three interventional fellows, two advanced trainees). Prior to rating, all raters attended a standardisation meeting where the lead investigator reviewed definitions, demonstrated example videos, and facilitated consensus building to ensure rating consistency. Definitions were provided on the online rating platform. A hit was defined correct if the diathermy effect touched the marked line. Otherwise, it was incorrect ( Fig. 1C ). Each deidentified video (and associated image) (e.g., Video 1 ) was evaluated by three independent raters who were blinded to endoscopist identity and selected randomly from the pool of seven raters using a custom online platform. This platform featured a video player and four buttons, 16 which allowed the rater to identify correct hit, incorrect hit, start and stop, and could be controlled using the keyboard with shortcuts to improve the precision of rating. When rating videos online, raters evaluated the following parameters: (1) correct hits, (2) incorrect hits, (3) percentage of the shape treated with electrosurgical hits, defined by amount of marked line treated based on the overview photograph, and (4) their subjective assessment of the quality of tip control, rated on a percentage scale, higher scores representing better performance. Once a video was rated, the rater could not go back to modify past ratings. Ratings were stored and exported for final analysis.

Raters anchored the subjective tip-control score on the following descriptors, presented in writing with example videos at the standardisation meeting and kept visible on the rating platform. A higher score reflected a stable scope position relative to the imprinted line, smooth, continuous tracking with minimal corrective movements, the tip rarely leaving the line, and uninterrupted, well-paced delivery of current pulses along the target. A lower score reflected an unstable scope position, frequent corrections, the tip repeatedly leaving the line, and irregular, often interrupted pulse delivery poorly aligned with the target.

#### Calculation of Parameters Related to Endoscopists Performance and Study Endpoints

To avoid time-related bias and accurately compare endoscopists’ skills, we standardised performance measures by adjusting for procedure time. Further analysis was conducted using standardised measures ( Box 1 ).

**Box 1 Standardised measures used in the reporting of tip control during the study**

- **Accuracy (%)** = correct hits/(correct hits + incorrect hits)
- **Speed (mm/min)** = shape length * percentage of treated shape/time (min)
- **Correct hit frequency (hits/min)** = number of correct hits (on marked line)/time (min)
- **Hit density (hits/cm)** = number of correct hits/shape length (cm)

### Statistical Analysis

The raw data file from the web application was analysed using R statistical software (version 4.4.1). Inter-rater reliability was assessed as absolute agreement using the intra-class correlation coefficient for partially crossed incomplete designs (two-way random effects model, ICC(A,k̂) with k̂ = 3) described by ten Hove et al. 17 Potential rating trends were assessed for each rater using linear regression models. In further analyses, the mean of the three independent ratings for each procedure was used, given the moderate-to-good inter-rater reliability of the measured metrics. The unit of analysis were procedures nested within endoscopists. We applied generalised linear mixed models (GLMMs) appropriate for repeated measures designs, with procedures modelled at the observation level and a random intercept for each endoscopist to account for within-endoscopist correlation. Because the outcomes differed in measurement scale, GLMM families were specified according to the theoretical support of each variable. This approach properly models within-subject changes (each endoscopist serving as their own control) while accounting for between-subject variability. 18 Pre- versus post-training comparisons included training status as a fixed effect and applying random intercepts to endoscopists. To identify predictors of improvement in subjective tip control, we analysed the change in subjective score (post-training minus pre-training) using a GLMM with baseline performance as a fixed effect and endoscopist as random effect, accounting for intra- and between-endoscopist variability. Descriptive statistics are given as estimated marginal means and corresponding confidence intervals. Linear trends were tested across experience levels (Expert, Fellow, Trainee) and shapes using polynomial contrasts. Pairwise comparisons between experience levels were conducted using “emmeans” with Tukey-adjusted contrasts. 19

## Results

### Participant Characteristics

Thirteen endoscopists participated (mean age 34.8 ± 4.7 years, 5 [38.5%] female). Participants were stratified by experience into three groups: one expert with >5 years independent practice and >500 lifetime EMRs, seven interventional fellows with mean 2 ± 0.8 years fellowship experience, and five trainees with mean 1.2 ± 0.4 years training experience. Five out of seven fellows had experience of ≥50 EMR procedures. Two trainees had performed between 20 and 50 EMRs, the rest had no EMR experience ( **Supplementary Table 1** ).

Each of the 13 endoscopists performed four pre- and four post-intervention procedures. Of these five endoscopists completed 12 baseline procedures and eight endoscopists completed 19 post-training procedures in less than 5 min, totalling 29.8% ( *n* = 31) of the 104 procedures.

### Primary Endpoint: Change in Subjective Tip-Control Scores Pre- and Post-Training

Post-training assessments were on average 14 (IQR: 7–20) days after the baseline evaluation. Overall, participants showed significant improvement in subjective tip control following two structured training sessions on the HAM-2 model. The subjective tip-control score increased by 12.3 points (95% confidence interval [CI]: 6.9–17.7; *p* < 0.001) post-training (the estimated marginal mean subjective score was 62.7 [95% CI: 54.0–71.4] and increased to 75.0 [95% CI: 67.8–82.2]). Improvements were most marked among fellows and trainees, while the single expert demonstrated minimal change in this outcome ( Table 1 , Fig. 2 ).

**Table 1:** Pre- and post-training performance metrics. Estimated marginal means, confidence intervals for pre- and post-training status groups and differences between groups were calculated with generalised linear mixed models.

Variable	Pre-training *n* = 52	Post-training *n* = 52	Difference	*p* -Value
Subjective				
Tip control	62.7	75	**12.3**	**<0.001**
	(54–71.4)	(67.8–82.2)	**(6.9–17.7)**	
Objective				
Correct hit ( *n* )	79.4	106.7	**27.3**	**<0.001**
	(68.4–90.5)	(91.9–121.5)	**(17.2–37.3)**	
Incorrect hit ( *n* )	5.7	6.4	0.7	0.332
	(4.3–7.2)	(4.8–8)	(–0.7–2)	
Accuracy (%)	92.6	93.9	1.3	0.085
	(90.6–94.5)	(92.2–95.6)	(–0.2–2.8)	
Hit frequency (hit/min)	17.2	23.2	**6.1**	**<0.001**
	(14.2–20.2)	(19.2–27.3)	**(3.7–8.5)**	
Hit density (hit/cm)	7.5	8.3	**0.8**	**0.018**
	(6.8–8.3)	(7.4–9.1)	**(0.1–1.4)**	
Speed (mm/min)	22.9	28.4	**5.5**	**<0.001**
	(19.5–26.3)	(24.2–32.7)	**(3.1–7.9)**	

**Fig. 2:**
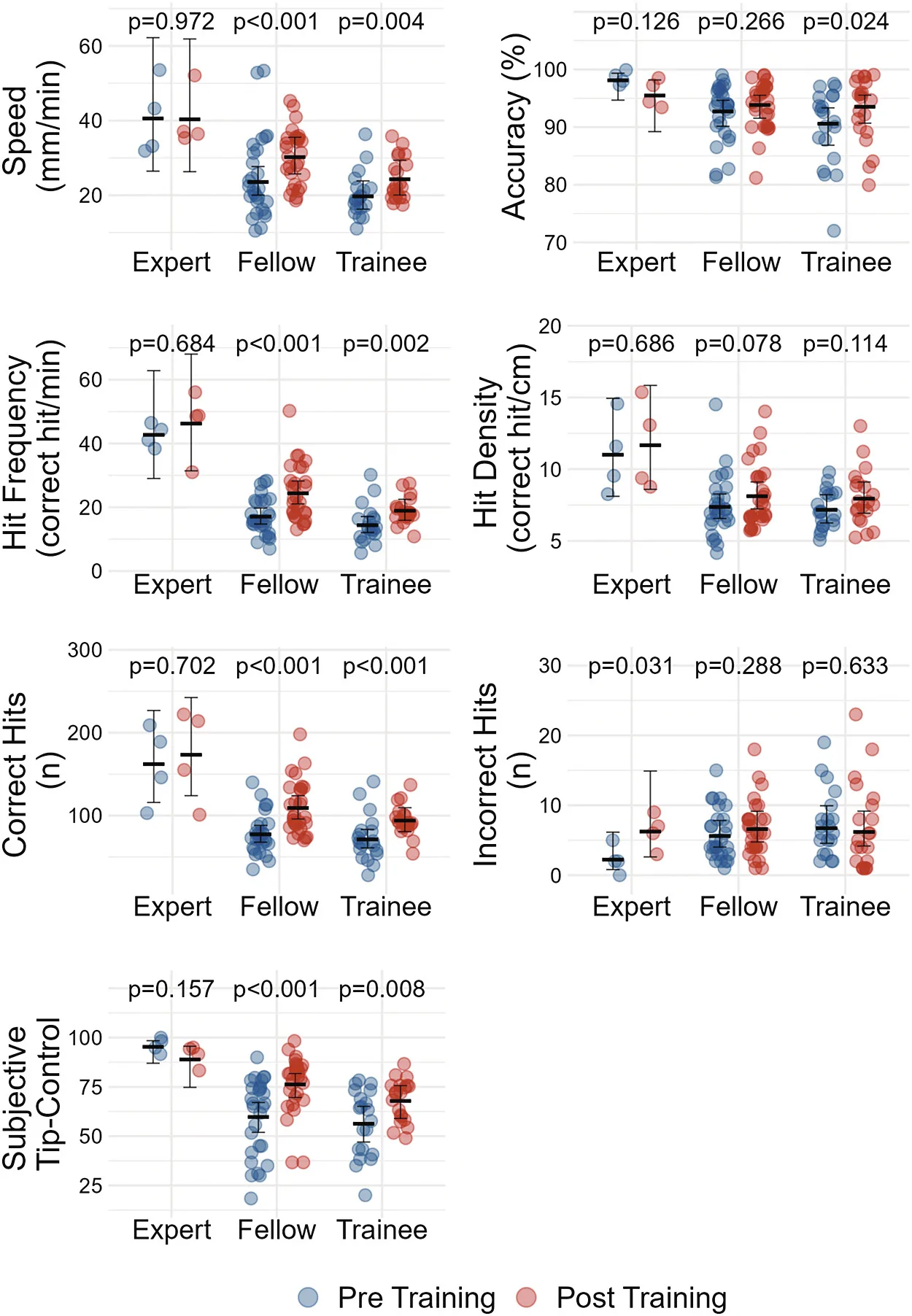
Pre- and Post-Training performance of endoscopists across different experience levels. Black bars indicate estimated marginal means and corresponding confidence intervals derived from generalised linear mixed models.

### Secondary Endpoints: Objective Performance Metrics Pre- and Post-Training

In addition to subjective improvements, objective performance metrics also demonstrated significant changes following structured training. The number of correct hits per procedure increased from a pre-training mean of 79.4 (95% CI: 68.4–90.5) to 106.7 (95% CI: 91.9–121.5) post-training ( Table 1 ), representing an increase of 27.3 correct hits (95% CI: 17.2–37.3; *p* < 0.001).

Speed improved by 5.5 mm/min (95% CI: 3.1–7.9; *p* < 0.001), from a pre-training mean of 22.9 mm/min (95% CI: 19.5–26.3) to a post-training mean of 28.4 mm/min (95% CI: 24.2–32.7). Similarly, the correct hit frequency increased by 6.1 hits/min (95% CI: 3.7–8.5; *p* < 0.001), from 17.2 (95% CI: 14.2–20.2) to 23.2 (95% CI: 19.2–27.3) hits/min after training.

Hit density improved significantly, increasing by 0.8 hits per centimetre (95% CI: 0.1−1.4; *p* = 0.018), from 7.5 hits/cm (95% CI: 6.8–8.3) to 8.3 hits/cm (95% CI: 7.4–9.1). Although accuracy demonstrated a modest upward trend from 92.6% (95% CI: 90.6–94.5) to 93.9% (95% CI: 92.2–95.6), this difference did not reach statistical significance (mean difference: 1.3%; 95% CI: −0.2 to 2.8; *p* = 0.085).

We additionally assessed whether the variable interval between assessments influenced the observed gains. Regressing each participant’s mean pre–post change on the elapsed interval (likelihood-ratio tests), a longer interval was associated with greater improvement in accuracy (+0.35% per day; *p* = 0.010), correct-hit frequency (+0.41 hit/min, *p* = 0.017) and speed (+0.44 mm/min, *p* = 0.004), but not in the subjective tip-control score ( *p* = 0.109).

### Secondary Endpoints: Stratification by Experience Level (pre-training, exploratory)

Expert procedures demonstrated superior baseline performance across all metrics compared to those of fellows and trainees. Specifically, the expert’s speed was 40.6 mm/min (95% CI: 26.5–62.2), compared to 23.6 mm/min (95% CI: 20.0–27.7) for fellows and 19.7 mm/min (95% CI: 16.3–23.8) for trainees ( *p* = 0.002 for linear trend). As only one expert was studied, these expert-versus-group comparisons are exploratory.

Accuracy followed a similar trend, with the expert achieving 98.1% (95% CI: 94.7–99.3) compared to 92.7% (95% CI: 90.2–94.7) among fellows and 90.6% (95% CI: 86.9–93.3) among trainees ( *p* = 0.003). Subjective tip-control scores were highest in the expert group at 95.3% (95% CI: 87.0–98.4), compared to 59.7% (95% CI: 51.9–67.1) for fellows and 56.3% (95% CI: 47.0–65.1) for trainees ( *p* < 0.001).

Endoscopists demonstrated similar accuracy and subjective tip‐control scores across different shape categories, suggesting consistent task difficulty ( **Supplementary Fig. 1** ). However, although hit frequency was similar across shapes, we observed higher speeds on longer shapes, which was achieved by applying hits at a lower density.

Following structured training, differences in accuracy between the experience groups were no longer significant. However, differences in speed and subjective tip control remained evident ( Table 2 ). Fellows and trainees were not statistically distinguishable on any metric, either before or after practice ( Table 2 ).

**Table 2:** Estimated marginal means (95% CI) and pairwise comparisons of performance metrics across experience levels (Expert, Fellow, Trainee) before and after unsupervised, structured training.

	Expert ( *n* = 1)	E vs. F *p* value	Fellow ( *n* = 7)	E vs. T *p* value	Trainee ( *n* = 5)	F vs. T *p* value	*p* -Value
** Pre-training procedures ( *n* = 52) **	*n* = 4		*n* = 28		*n* = 20		
Correct hit ( *n* )	162.1	**<0.001**	77.3	**<0.001**	71.2	0.708	**<0.001**
	(115.8–226.8)		(67.9–88.1)		(61.0–83.2)		
Incorrect hit ( *n* )	2.2	0.208	5.6	0.114	6.7	0.765	**0.046**
	(0.8–6.2)		(4.0–7.8)		(4.6–9.9)		
Accuracy (%)	98.1	**0.036**	92.7	**0.010**	90.6	0.510	**0.003**
	(94.7–99.3)		(90.2–94.7)		(86.9–93.3)		
Speed (mm/min)	40.6	0.052	23.6	**0.007**	19.7	0.341	**0.002**
	(26.5–62.2)		(20.0–27.7)		(16.3–23.8)		
Correct hit frequency (hit/min)	42.7	**<0.001**	17.1	**<0.001**	14.4	0.298	**<0.001**
	(29.0–62.8)		(14.7–19.8)		(12.1–17.1)		
Correct hit density (hit/cm)	11.0	**0.042**	7.4	**0.032**	7.2	0.952	**0.012**
	(8.1–14.9)		(6.6–8.3)		(6.3–8.2)		
Tip control (subjective)	95.3	**<0.001**	59.7	**<0.001**	56.3	0.837	**<0.001**
	(87–98.4)		(51.9–67.1)		(47–65.1)		
** Post-training procedures ( *n* = 52) **	*n* = 4		*n* = 28		*n* = 20		
Correct hit ( *n* )	173.3	**0.031**	109.1	**0.003**	93.9	0.307	**0.001**
	(124.0–242.4)		(95.9–124.1)		(80.6–109.4)		
Incorrect hit ( *n* )	6.2	0.993	6.6	1.000	6.2	0.969	0.985
	(2.6–14.9)		(4.7–9.1)		(4.2–9.2)		
Accuracy (%)	95.5	0.793	93.8	0.743	93.5	0.979	0.462
	(89.2–98.2)		(91.5–95.5)		(90.7–95.5)		
Speed (mm/min)	40.3	0.431	30.2	0.085	24.3	0.199	**0.034**
	(26.3–61.9)		(25.7–35.5)		(20.0–29.4)		
Correct hit frequency (hit/min)	46.2	**0.007**	24.4	**<0.001**	18.9	0.073	**<0.001**
	(31.4–68.0)		(21.1–28.2)		(15.9–22.5)		
Correct hit density (hit/cm)	11.7	0.075	8.1	0.063	7.9	0.969	**0.024**
	(8.6–15.8)		(7.2–9.1)		(6.9–9.1)		
Tip control (subjective)	88.9	0.2	76.2	**0.038**	67.8	0.243	**0.014**
	(74.7–95.6)		(69.6–81.7)		(58.9–75.6)		

Values represent estimated marginal means with 95% confidence intervals from generalised linear mixed models. Pairwise comparisons between experience groups were conducted using emmeans with Tukey-adjusted contrasts. **E versus F** = Expert versus Fellow, **E versus T** = Expert versus Trainee, **F versus T** = Fellow versus Trainee. The rightmost column shows *p* -values for linear trends across experience levels, obtained from polynomial contrasts conducted separately for pre- and post-training conditions. Bolded *p* -values indicate statistical significance at *p* < 0.05.

### Baseline Performance Metrics Predicting Change in Subjective Tip Control

Baseline performance was associated with subsequent improvement in subjective tip control following structured training. Endoscopists with lower baseline accuracy and speed demonstrated the greatest gains ( **Supplementary Fig. 2** ). In multivariable analysis, lower baseline accuracy (β estimate: −1.61, 95% CI: −2.66 to −0.56; *p* = 0.003) and lower baseline speed (β: −0.84, 95%CI: −1.61 to −0.06; *p* = 0.035), were independent predictors of improvement in subjective tip control ( Table 3 ).

**Table 3:** Association of baseline performance measures with change in subjective tip control after training. Univariable and multivariable generalised linear mixed model analyses examined baseline performance measures as predictors of change in subjective tip control following training. Estimates are presented as beta values with 95% confidence intervals (CIs). Negative estimates denote an inverse association with subsequent improvements in subjective tip control.

	Predictor	Univariable			Multivariable		
Outcome	Variable at baseline	Estimate (β)	95% CI	*p* -Value	Estimate (β)	95% CI	*p* -Value
Change in subjective tip control	Accuracy (%)	−2.08	−3.06 to −1.11	<0.001	***−1.61***	− ***2.66 to −0.56***	***0.003***
	Hit frequency (hit/min)	−1.19	−1.79 to −0.59	<0.001	−0.01	−1.01 to 0.99	0.987
	Hit density (hit/cm)	−1.36	−4.39 to 1.67	0.379	− ***0.84***	***−1.61 to −0.06***	***0.035***
	Speed (mm/min)	−1.14	−1.72 to −0.56	<0.001			

### Inter-Rater Reliability

Seven raters evaluated a total of 31.05 h of procedural videos. Raters assessed a median of 52 videos (IQR: 46–56), with a median total viewing duration of 5.16 h (IQR: 4.57–5.35). The intra-class correlation coefficient for subjective impression of tip control, the number of correct and incorrect hits was 0.74 (95%CI: 0.61–0.87), 0.82 (0.73–0.90), and 0.71 (0.58–0.83), respectively, indicating moderate to good absolute agreement across the three raters per video. Furthermore, there was no clear trend across raters to rate a video better or worse over time, as a linear model did not show a trend across ratings for number of correct and incorrect hits, or subjective tip-control score ( **Supplementary Fig. 3** ).

## Discussion

This study evaluated the HAM-2 ex vivo STSC model as a low-cost platform for deliberate self-practice and assessment of endoscopic tip control. Unsupervised, structured practice using the model, even in this cohort of relatively advanced endoscopists, led to improvements in subjective and objective measures of tip control. Furthermore, the model effectively stratifies endoscopists by their experience level based on both subjective and objective performance metrics. These findings suggest that the HAM-2 model is a reproducible framework for assessing and practicing a specific component of endoscopic skill, that is, tip control. Although the physical model is suitable for repeated local self-practice, the current manual multi-rater assessment process is labour-intensive and not yet scalable for routine use.

The primary endpoint was the change in subjectively assessed tip control following structured self-practice. Participants demonstrated significant improvement in subjective scores, particularly fellows and trainees. Notably, the expert endoscopist demonstrated no improvement. With a single expert, this cannot distinguish a true ceiling effect from individual variation, and no plateau can be defined from the present data. These results are consistent with the hypothesis that unsupervised self-practice on a focused motor task relevant to endoscopic practice can enhance tip control, particularly in endoscopists with lower baseline proficiency. However, the intervention evaluated here should be interpreted as structured, unsupervised self-practice rather than supervised training in the full educational sense. Individualised feedback was deliberately omitted in order to isolate the effect of the model itself and avoid additional variability related to trainer style, feedback content, and participant response to feedback.

We chose subjective scoring as our primary assessment method because it directly evaluates the core clinical skill under investigation, continuous fine-motor tip control. In clinical practice, tip control depends on the integration of wheel deflection, torque, shaft manipulation, tip stability, smoothness, and task effectiveness. These components are usually judged holistically by supervising endoscopists during live procedures. Nevertheless, subjective scoring has important limitations, including observer bias, variability in expectations, and limited mechanistic transparency. While we acknowledge that fully objective, performance-based metrics would be ideal, no validated objective standard currently exists for rating tip control. To mitigate this concern, we used a blinded, multi-rater design in which each participant’s performance was assessed by three independent evaluators in a random order. This approach yielded reproducible scores, supported by a good intra-class correlation coefficient. For context, inter-observer agreement for the Paris classification is consistently only fair-to-moderate (κ ≈ 0.19–0.42) even after training, whereas our ICC for subjective tip control was 0.74. We acknowledge ICC and κ are not interchangeable. 20 21 Our concurrent computer-vision work (HAM-AI) aims to progressively replace this subjective score with objective kinematic metrics derived from the same recordings.

Importantly, objective metrics mirrored these subjective findings. Participants demonstrated notable improvements in speed, correct hit frequency, and hit density after two practice sessions, whereas accuracy did not change. This may suggest a trade-off between speed and precision during task performance (pressure to complete the task within 5 min) or the possibility that accuracy, as measured in this context, requires longer, more extensive practice to meaningfully improve.

The model’s ability to stratify endoscopists by experience level at baseline indicates good construct validity. The expert consistently outperformed fellows and trainees across key performance metrics, including speed, accuracy, and subjective tip control. After practice, the significant reduction in performance differences for accuracy between groups shows that targeted practice may allow less experienced endoscopists to close the gap in this domain. Potentially, by isolating tip control from other procedural complexities (e.g., injection technique, specific resection methods or haemostasis) or aspects of endoscopy not requiring fine tip manipulation, trainees focused on developing the fine motor skills at the core of endoscopic manoeuvres. By contrast, the trend across experience levels for speed and subjective tip control remained significant ( Table 2 ), highlighting the need for more than two practice sessions to develop efficiency and smoothness of endoscopic movements. Fellows and trainees, however, were not statistically separable on any metric, indicating that the model resolves the expert–non-expert distinction rather than finer experience gradations, consistent with its isolation of a specific fine-motor competency that is not a simple function of cumulative procedural volume.

Analysis of changes in subjective scores pre- versus post- training suggests that lower baseline accuracy and speed were associated with greater improvements in subjective tip control following structured self-practice. These findings indicate that participants with lower initial performance may benefit more from repeated practice using the HAM-2 model. Furthermore, this observation supports the potential utility of the model as an unsupervised practice tool particularly suited to early stages of skill development, where gains in fine motor control may be more readily achieved. However, in this single-arm, pre-/post-design, part of this inverse association between baseline performance and improvement is expected from regression to the mean and a ceiling effect among higher performers; this finding should therefore be regarded as exploratory and hypothesis-generating.

Few prior studies have explicitly isolated tip control as a distinct competency for training and assessment in gastrointestinal endoscopy. Riek et al. 22 introduced a colonoscopy tip-control training device that differentiated experience levels and demonstrated that novices could improve their performance to match that of experienced operators following training. Similarly, Kamolvisit et al. reported comparable findings using the Varix Trainer, which focused on scope navigation rather than fine motor control. 23 Both studies, while valuable, differ fundamentally from our approach. Specifically, they assessed the ability to bring the target into the endoscopic field of view but did so at distances (e.g., 30 and 20 mm) that do not reflect the spatial precision required in actual endoscopic procedures. Notably, torque application was restricted in some assessments of the Varix Trainer, further limiting the model’s resemblance to real-life endoscopy.

In contrast, our model evaluates tip control through a clinically relevant task—STSC applied to a defined shape—which demands continuous focus, coordination, and fine motor precision. The task model preserves the full range of endoscopic movements, including tip deflection, torque application, and shaft manipulation—all essential components of real-world endoscopy. We propose that this more closely replicates the demands of procedures such as STSC or endoscopic submucosal dissection, rather than shorter, less continuous tasks such as submucosal injection or clip placement. Our model requires higher precision: a deviation of just 1 mm reduces accuracy. While tip control encompasses multiple dimensions, we believe the HAM-2 framework captures a core aspect of clinically meaningful tip-control performance. Its emphasis on sustained, fine motor control aligns with both the demands of basic endoscopy and advanced therapeutic procedures. The model’s reliance on a genuine electrosurgical task performed with a clinical gastroscope and snare further supports its face validity.

Our findings build on earlier work demonstrating that STSC performance correlates with endoscopist experience in vivo 13 and ex vivo. 14 The HAM-2 model refines the HAM-1 14 design with standardised tasks and objective, blinded scoring, overcoming limitations related to inter-rater variability and lack of reproducibility. Compared to expensive and logistically challenging training systems—such as VR simulators or live animal models—the HAM-2 model provides a cost-effective alternative requiring only standard endoscopic tools. Excluding the training box and endoscope, which are typically already available in endoscopy training units, the marginal consumable cost per procedure is well under €5. Its scalability, however, is currently limited by the assessment rather than the platform: manual assessment of the study recordings required 31.05 cumulative rater-hours, the principal non-consumable implementation cost of the present process.

A major strength of this study is its rigorous design, using blinded, independent raters and standardised scoring criteria.

However, several limitations warrant consideration. First, the single-centre design and the inclusion of a single expert limit the generalisability of our findings, particularly with regard to defining a robust expert benchmark. The study was conceived as a pilot validation of the HAM-2 task and scoring framework and was deliberately confined to the unit in which the model was developed. A multicentre validation study with multiple experts is planned and will specifically address expert standards, and the subgroups of endoscopists that would benefit the most from such a training. Second, although participants showed improvements in subjective and objective performance measures, this single-arm study, with a short (two-session) training period, was not designed to assess whether these improvements translate into enhanced clinical outcomes. It remains unclear how many sessions are required to achieve mastery of tip control. Transfer to other endoscopic tasks therefore remains unproven and requires prospective validation using independent task-based and patient-based performance endpoints. Third, the current model is two-dimensional and does not require retroversion, difficult positioning, or marked axial rotation. A three-dimensional version is under development to address this limitation. Fourth, cleaning episodes were not systematically recorded. Because lens washing was switched off to prevent fluid accumulation on the ham surface, cleaning partly reflected the experimental setup and operator style rather than competence alone. Fifth, the rater workload described above, although feasible in a research setting, may limit the practicality of manual assessment in clinical training programmes; future studies should explore automated or semi-automated assessment systems to reduce rater burden. Sixth, the present study did not evaluate a feedback-based educational intervention: participants did not receive their scores, individualised coaching, or corrective feedback between sessions. The assessment outputs quantify what was achieved but do not identify the underlying technical cause of poor performance, such as inappropriate wheel control, torque, shaft manipulation or scope instability. HAM-2 should therefore be regarded at present as a platform for repeated task-specific practice and performance measurement rather than as a stand-alone feedback system. Finally, as a single-arm study without a non-practising control group, we cannot exclude that part of the observed improvement reflects interim clinical endoscopy exposure between assessments rather than self-practice on the model alone.

## Conclusions

The HAM-2 ex vivo model demonstrated preliminary construct validity (exploratory, given the single expert) and good inter-rater reliability as a tool for assessing endoscopic tip control. Structured self-practice on the model significantly improved both subjective and objective markers of tip control, particularly in lower-performing endoscopists. Given its affordability, simplicity, and reproducibility, the HAM-2 model represents a promising adjunct to existing endoscopic training methods, provided the assessment burden can be reduced.

## Data Availability

Data are available upon reasonable request to authors.

## Declaration of GenAI Use

Generative AI was used solely for language editing. All AI-edited text was subsequently reviewed and approved by the authors. Generative AI was not used to develop the analytical pipeline, create images, interpret the results, or generate the scientific or intellectual content of this article.

## Supplementary Material

Ergänzendes Material Supplementary Material
