# Supplementary material for: A Novel ex vivo Snare Tip Soft Coagulation Model for Deliberate Self-Practice and Assessing Endoscopic Tip-Control Competency: The HAM-2 Study
Source: Endosc Int Open. 2026 Sep 23;14:a29536394. doi: 10.1055/a-2953-6394 (PMC13643895; doi:10.1055/a-2953-6394)

Supplementary material: A Novel Ex-Vivo Snare Tip Soft Coagulation Model for Deliberate Self-Practice and Assessing Endoscopic Tip-Control Competency: The HAM-2

Study

Supplementary Table 1. Demographic characteristics of participant endoscopists.

	Expert	Interventinal Fellow	Trainee
**Years of independent practice/fellowship/training (mean, sd)**	>5	2±0.8	1.2±0.4
**smsa level**			
**4+**	1	2	
**4**		3	
**3**		2	1
**1**			4
**Colonoscopy experience**			
**>1000**	1	6	
**500-1000**		1	2
**<500**			3
**EMR experience**			
**>200**	1	1	
**50-200**		4	
**20-49**		2	2
**<20**			3

Supplementary Figure 1. Performance metrics across different shapes.

Individual observations are plotted for each endoscopist, performing on each shape separated by pre- and post-training status. To evaluate the monotonic effect of shape level, we fitted a generalized linear mixed-effects model per variable, with a shape * training status (pre-post) interaction (including endoscopists as a random effect) and used orthogonal polynomial contrasts to assess a possible linear trend.

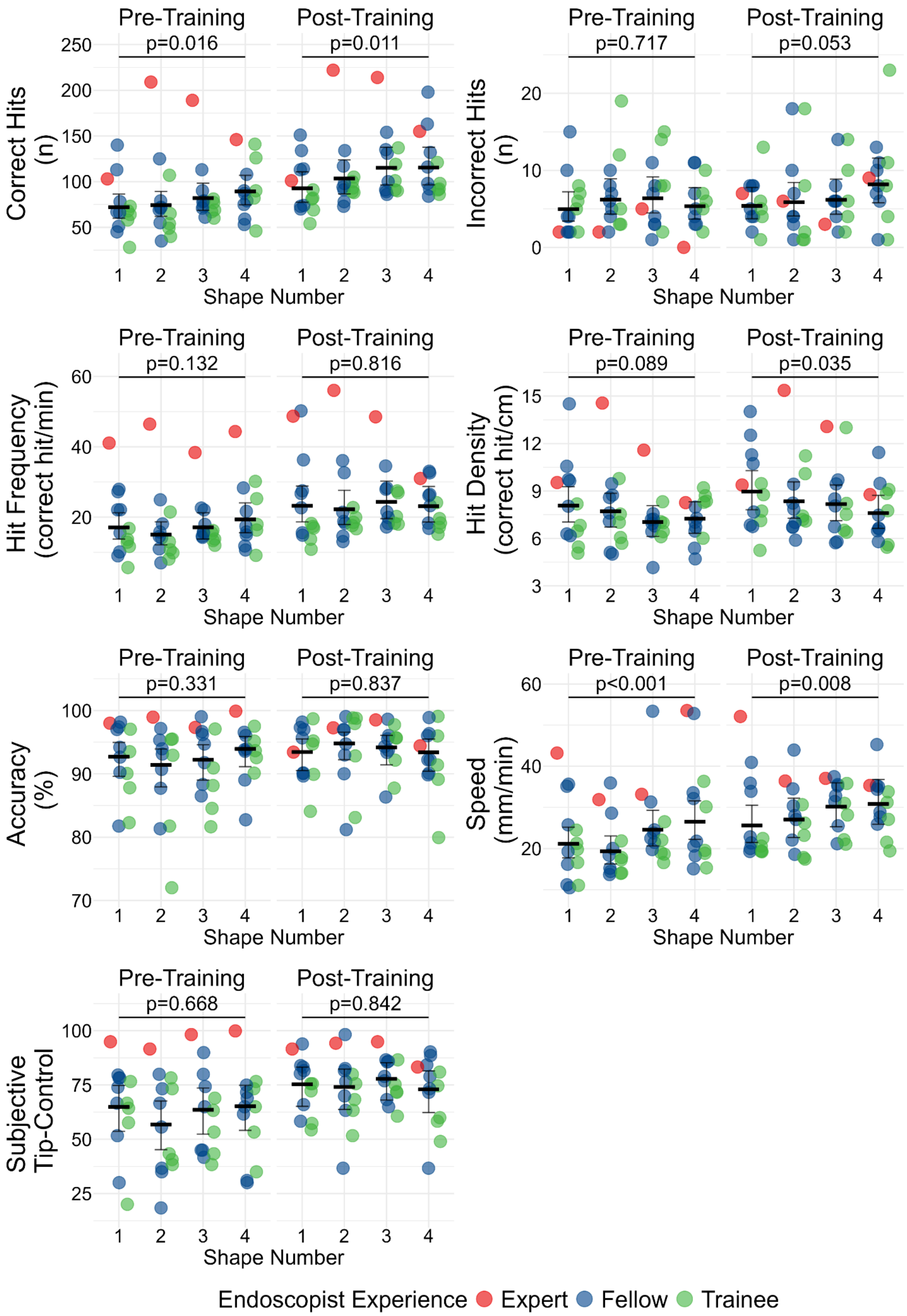
Correct Hits (n)
Pre-Training p=0.016
Post-Training p=0.011
Incorrect Hits (n)
Pre-Training p=0.717
Post-Training p=0.053
Hit Frequency (correct hit/min)
Pre-Training p=0.132
Post-Training p=0.816
Hit Density (correct hit/cm)
Pre-Training p=0.089
Post-Training p=0.035
Accuracy (%)
Pre-Training p=0.331
Post-Training p=0.837
Speed (mm/min)
Pre-Training p<0.001
Post-Training p=0.008
Subjective Tip-Control
Pre-Training p=0.668
Post-Training p=0.842
Shape Number
Endoscopist Experience
Expert
Fellow
Trainee

Supplementary Figure 2. Pre- and Post-Training performance of individual endoscopists.

Arrow tails and arrow heads represent mean accuracy and speed for individual endoscopists, pre- and post-training, respectively. Blue and red dashed lines on both axes represent group mean, pre- and post-training. Red dot-dashed curve represents 95% confidence line under that there is predicted improvement in subjective tip-control after training and is derived from the multivariable generalized linear mixed model.

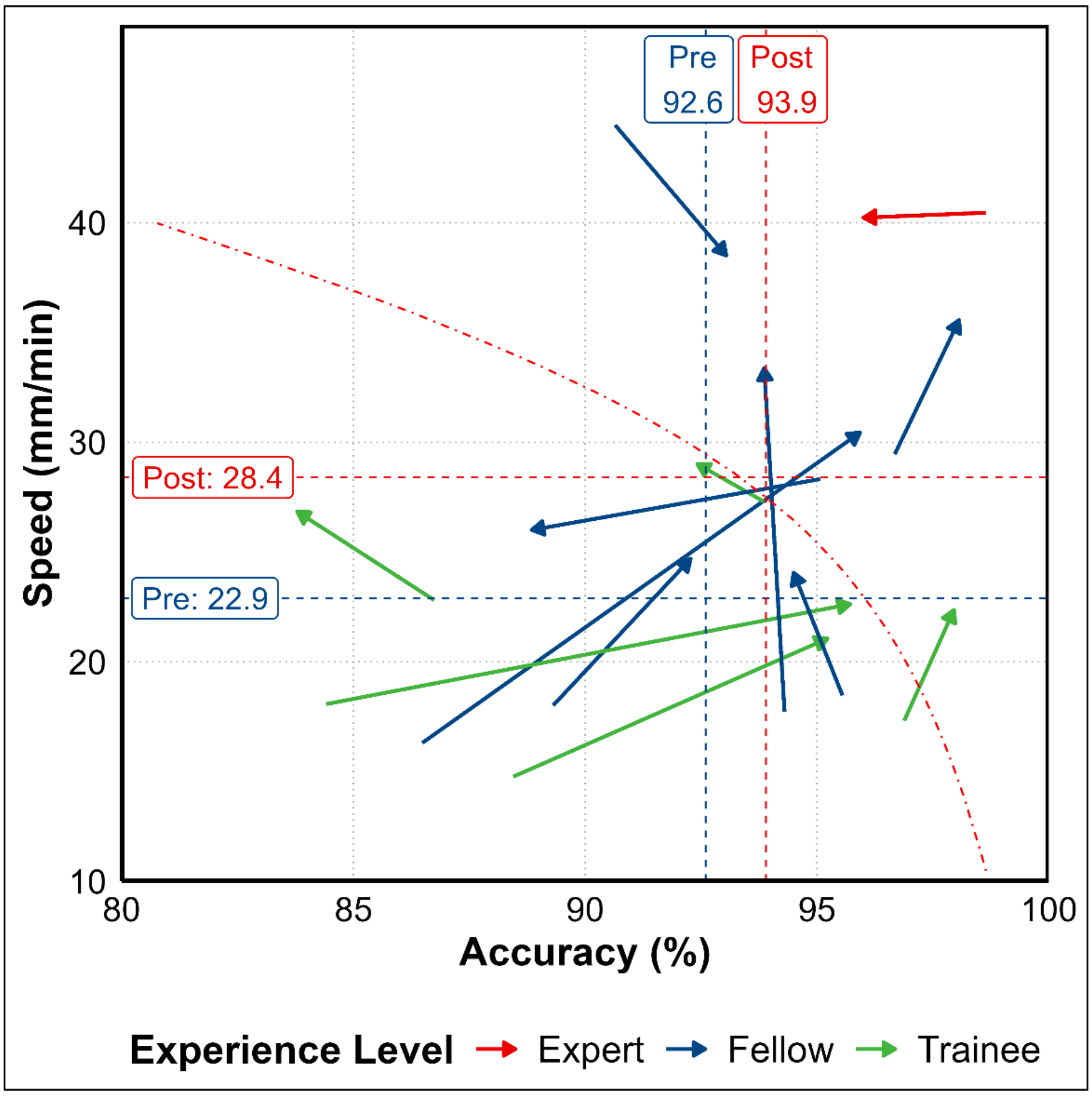

Supplementary Figure 3. Ratings over time by rater with linear regression trends for each variable.

For each rater and response variable - Correct Hit, Incorrect Hit, and Tip-control (Subjective) - ratings are plotted against date-time rank of rating. Linear regression lines were fitted, each panel displays R^2 and p-values to indicate the strength and significance of potential trends.

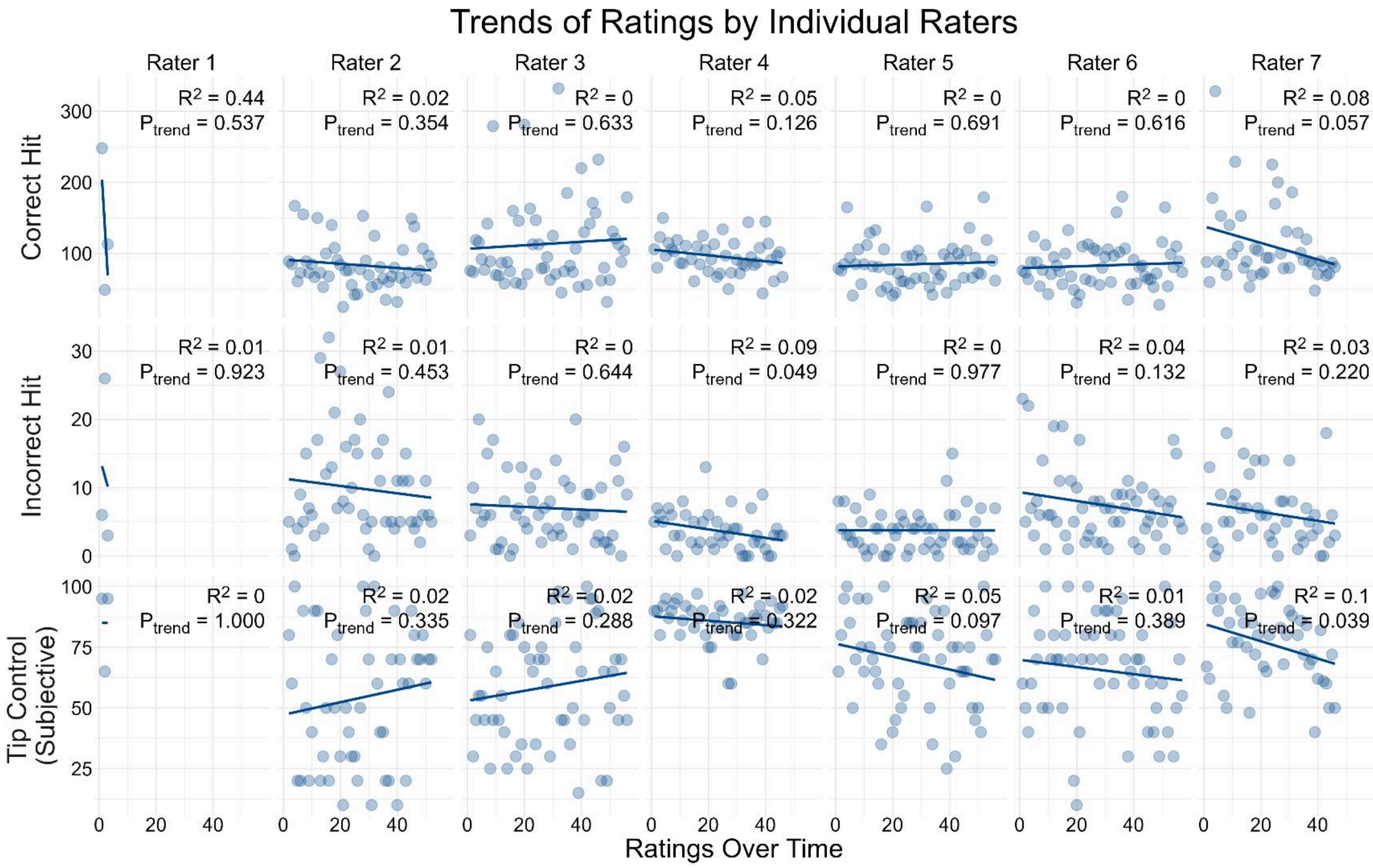

Supplement: Supplementary file 1 [file 10-1055-a-2953-6394_29609873.pdf]
